# Non-green influencers promoting sustainable consumption: Dynamic norms enhance the credibility of authentic pro-environmental posts

**DOI:** 10.3389/fpsyg.2023.1112762

**Published:** 2023-02-09

**Authors:** Ágnes Buvár, Ágnes Zsila, Gábor Orosz

**Affiliations:** ^1^Institute of People-Environment Transaction, ELTE Eötvös Loránd University, Budapest, Hungary; ^2^Institute of Psychology, Pázmány Péter Catholic University, Budapest, Hungary; ^3^Université d'Artois, Unité de Recherche Pluridisciplinaire Sport Santé Société, Sherpas, Liévin, France

**Keywords:** authenticity, credibility, dynamic norm, expert opinion, social media influencer, sustainable consumption

## Abstract

Social media influencers can raise awareness for sustainability, and establish norms related to a more sustainable lifestyle. Although non-green influencers can reach a wider audience, they might face credibility issues when communicating about sustainable consumption. In the present 2×2 online mixed method experiment (*N* = 386), we explored the effect of two credibility-enhancing strategies (authenticity vs. referring to experts) and the presence (vs. lack of) of dynamic norms (information about how other people's behavior is changing over time) on the perceived credibility of the post. Results indicated that referring to expert opinion enhanced perceived post credibility. However, if an authentic message was combined with dynamic norms, it reduced the frequency of mentions of the lack of credibility. Both credibility measures were positively associated with the persuasiveness of the message. These findings can contribute to the growing literature of credibility-enhancing strategies and dynamic norms. The study also provides practical suggestions for non-green influencers on effective communication of sustainable consumption.

## 1. Introduction

### 1.1. Sustainability messages from green and non-green influencers

Social media can be used to raise awareness for and rapidly disseminate information about sustainability to younger people who are less approachable *via* traditional media (Johnstone and Lindh, 2018; Chan et al., 2020). The role of social media influencers—content providers with considerable follower base, perceived as opinion leaders (Uzunoglu and Misci Kip, 2014; Evans et al., 2017)—in promoting sustainable lifestyle and eco-consciousness constitutes an emerging research area in the field of sustainability communication. Previous research was mainly concerned about green influencers or promoting sustainable products and services. For instance, Pittman and Abell (2021) found that green influencers with less followers are more persuasive in promoting sustainable products. Kapoor et al. (2022) examined how argument quality affected the persuasiveness of eco-friendly hotel recommendations and found that attribute-based messages that provided objective and rational information were more persuasive than simple recommendations.

Some social media influencers are experts in specific areas like beauty, fitness, or sustainability, while others produce entertaining content or document their everyday life such as lifestyle influencers (Lou and Yuan, 2019; Campbell and Farrell, 2020). Besides green influencers whose profile and activities are centered around sustainability; non-green influencers can also encourage sustainable consumption. They can reach a larger, less eco-conscious audience. Moreover, as their lifestyle is usually not perfectly green, they do not generate reactance in their audience by pointing out the difference between the users' ideal and actual sustainable selves (Jin et al., 2019). On the other hand, posts about sustainability from a non-green influencer can raise concerns regarding credibility as they are not perceived as experts in the field, nor do they showcase a sustainable lifestyle that could make them trustworthy and authentic sources about sustainability. Moreover, when people's expectations are not met (i.e., when a non-green influencer posts about sustainability), it is likely that information processing will be more complicated that can consequently lower the credibility of the message (Marsh and Yang, 2021). Thus, we aimed to investigate how non-green influencers can communicate sustainable messages such as the reduction of single-use plastic in an effective and credible way by manipulating two variables: the type of credibility-enhancing strategy used in the social media post and the presence of dynamic norms.

### 1.2. Expert opinion argument

Expertise is one of the core components of the communicator's credibility that affects the persuasiveness of a message in a way that higher perceived expertise is associated with higher perceived source credibility, which in turn leads to greater acceptance of the persuasive message (Ohanian, 1990). Moreover, when the source is an expert, it may motivate recipients to take the message more seriously (Kruglanski et al., 2005). Experts can “confer authority” on other sources (Kruglanski et al., 2005). Indeed, the use of expert opinion consists of citing someone's opinion who is an authority (expert) in the field, similarly to the use of literature references in research papers (Hoeken and Hustinx, 2003). Previous findings indicated that expert opinion is as persuasive as statistical and causal evidence, and more persuasive than anecdotal evidence (Hoeken and Hustinx, 2003). Consequently, we hypothesized that including an expert's opinion in a social media post can positively affect the credibility of the message as it triggers the authority heuristic (“expert statements can be trusted”) (Sundar, 2008). Message credibility is defined as the perceived veracity of the content and focuses on how the content of the communication is perceived (Appelman and Sundar, 2016).

### 1.3. The role of authenticity

Social media influencers are usually considered authentic and trustworthy sources (Djafarova and Rushworth, 2017). Trustworthiness constitutes another key factor of source credibility: higher perceived trustworthiness leads to higher perceived credibility, which in turn results in better persuasiveness (Ohanian, 1990). On the other hand, along with originality and spontaneity, trustworthiness is an integral part of authenticity (Enli, 2015). Authenticity includes what is perceived to be real and true regarding the source, the message, or the interaction; it also concerns the perceived gap between the influencer's claimed and real identities (Lee, 2020). As authenticity overlaps with credibility, if an influencer increases the authenticity of the post, and thereby, the credibility of the message, this could possibly lead to better persuasiveness.

According to self-determination theory, authenticity is associated with intrinsic (authentic) motivation that stems from the individual's own interests and values rather than being the result of external pressures (Ryan and Deci, 2000). As users are generally skeptical about green claims (Matthes and Wonneberger, 2014), if a non-green influencer posts about sustainable consumption, inauthenticity can trigger inferences about the influencer's ulterior, extrinsic motives such as posting about sustainability to gain more followers or improve their image by promoting a trending topic. Influencers might adapt different strategies to be perceived as authentic such as using personal language or having a unique style of communication expressed in the consistent choice of text and images (Abidin and Otis, 2016). Moreover, influencers can convey a more authentic image if they express how passionate they are about a topic (Audrezet et al., 2020). Thus, we propose that if the influencer expresses passion for sustainability while using their own communication style consistently, it can increase the authenticity of the post that in turn leads to higher message credibility.

### 1.4. Dynamic norms

Social information, including social norms can be effective tools in influencing one's pro-environmental behavior (Fanghella et al., 2019; Bruchmann et al., 2021). Moreover, social norms can be formed through exposure to media (Rimal and Storey, 2020). Therefore, social media influencers could be used to shape the norms of younger users that would promote a more sustainable lifestyle. However, when an unsustainable behavior is accepted in the society (i.e., meat consumption), referring to the norm (i.e., most people regularly eat meat) will probably not encourage people to change their behavior in a more sustainable direction. Instead, it can serve as a rationale to maintain unsustainable behavior (“if the majority does it, there is nothing wrong with it”). In that case, it is more effective to highlight the change of norm over time instead of promoting the actual norm (Sparkman and Walton, 2017). Indeed, previous research found that dynamic norms are effective in promoting sustainable behavior when the unsustainable behavior is still widely accepted in the society (Sparkman and Walton, 2017; Loschelder et al., 2019; Sparkman et al., 2021). Moreover, referring to the increasing number of individuals who think sustainable consumption is important can trigger bandwagon heuristic (“if others think it is important, I should think so, too”) that leads to increased message credibility (Sundar, 2008). Consequently, we propose that including dynamic norms in an influencer post that contains a sustainability message can increase the credibility of the post compared to the same post without dynamic norms.

### 1.5. Overview of the present research

The aim of the current study is to explore how non-green influencers can communicate effectively about sustainable consumption. It appears that the lack of credibility can play a crucial role in this mechanism, since non-green influencers are not sustainability experts, and the authenticity of such communication is also questionable as it may not meet the audience's expectations. Therefore, we tested two different credibility-enhancing strategies to examine which one has a more powerful positive effect on post credibility. We also examined whether dynamic norms could strengthen the credibility increase of the authenticity and expert strategies. Finally, based on previous empirical findings (Martínez-López et al., 2020), we also tested whether message credibility is positively related to the perceived persuasiveness of the message.

To examine these effects, we chose the topic of reducing single-use plastics such as PET bottles as it represents a considerable environmental issue (Chen et al., 2021). In summary, we posited that if a non-green influencer refers to an expert's opinion about the consequences of single-use plastics or they apply strategies to increase the authenticity related to sustainability, these strategies could have a positive impact on the credibility of a social media post encouraging the reduction of single-use plastics, especially PET bottles. Moreover, we also examined whether the presence of dynamic norms reinforce the positive effect of the two credibility-enhancing strategies.

## 2. Method

### 2.1. Participants and procedure

We designed a 2 (credibility-enhancing strategy: expert opinion vs. authenticity) × 2 (presence of dynamic norm: control vs. dynamic norm) online experiment. The study was approved by the Institutional Ethics Committee of the first author's university (no.2021/266-4). The research protocol was in accordance with the Declaration of Helsinki. All participants provided informed written consent at the beginning of the study.

According to the a-priori power analysis, a sample size of 387 persons was required to detect a small effect ($\eta_{\text{p}}^{2}$ = 0.02) at 0.8 power. Thus, we aimed for a sample of 400 respondents. However, after cleaning the database, the final convenience sample comprised 386 participants (*M*_age_ = 22.0 years, SD_age_ = 3.88, 71.9% female): 73.1% of them are BA students, almost half of them (47.2%) resides in the capital city, while an additional 21.8% lives in a smaller town, and 16.6% lives in a county capital.

### 2.2. Experimental stimuli

A fitness/lifestyle influencer was chosen for this experiment with 207 000 followers (11/2022). He is well-known to a larger audience due to his reality show appearances, and his posts are regularly reviewed by the national media. Although he is not a typical climate activist, he regularly posts pro-environmental content including participation in climate demonstrations or cooperation with the local Greenpeace organization. All participants were familiar with the influencer, and 12.7% claimed that they followed him on a social media platform.

To increase ecological validity, we used an already published photo of the lifestyle-fitness influencer, and we selected extracts of his social media posts about sustainability (i.e., reference to grandchildren, the David Attenborough documentary or partying) which we used to create posts that correspond to the experimental manipulations.

The main message of all four Instagram posts was about reducing PET plastic usage: “*Don't buy PET bottles, drink from a glass bottle instead!”*. In the expert opinion condition, we used the following main argument: “*I saw in the @davidattenborough documentary on Netflix that PET bottles often end up in the water as rubbish, and this is bad for the seas, the oceans and the animals living in it”*. Dynamic norms were presented as: “*More and more people care about the PET pollution every day”*.

In the authenticity condition, we used the following main argument: “*I want to hear my grandchildren saying that grandpa [name of the influencer] did not pollute our Planet with plastic sh@t!”*. Dynamic norms were presented as: “*More and more people join us in the PET-free party*”.

### 2.3. Measures

#### 2.3.1. Post credibility

We adapted a credibility scale from Cotte et al. (2005). Three items (α = 0.935) measured the perceived credibility of the post (“*The image and the text of the post is believable.”*, “*This post is truthful.”*, “*This post is realistic.”*) rated on a 5-point Likert (1:Strongly disagree) to (5:Strongly agree).

#### 2.3.2. Lack of credibility

We also formulated an open-ended question *(”If you were to talk about the post to one of your friends, what would you say?”*). The question was posed after the quantitative measures including the credibility scale. Answers to this question were coded based on whether they mentioned that the post is not credible (1), or they did not mention anything about the post's credibility (0). Coding was executed by two independent coders who achieved acceptable interrater agreement (weighted Kappa = 0.726). Disagreements were discussed until coders reached consensus. Typical verbatims that were coded as mentioning lack of credibility included “*not credible”*, “*hypocritical”*, “*he acts as if he were eco-friendly”* or “*he does not act in line with what he writes”*.

#### 2.3.4. Persuasiveness

Persuasiveness was assessed with one question (“*To what extent do you think that the post is persuasive?”*). Answers were given on a slider from 0 (very unlikely) to 100 (very likely).

### 2.4. Statistical analysis

Statistical analysis was performed using SPSS 27. First, descriptive statistics were calculated. Subsequently, we examined the internal consistency of the measures used in this study. Second, we examined the equal distribution of the control and demographic variables across experimental groups. Third, we conducted a two-way ANOVA to test whether post credibility is affected by the manipulated variables, and a binary logistic regression to test whether the lack of credibility of the post – a variable derived from the qualitative data – is affected by the manipulated variables. Finally, a hierarchical regression analysis was conducted to examine the predictive power of the two credibility variables on the persuasiveness of the post.

## 3. Results

We conducted several one-way ANOVAs to test the equal distribution of age, familiarity with the influencer, attitudes toward plastic and practices related to plastic waste avoidance before comparing the effect of the experimental stimuli across experimental groups. Results indicated equal distribution (all *p* > 0.480). In the following step, we conducted χ^2^ tests to test the equal distribution of gender, education, and place of residence. Results indicated equal distribution regarding education and place of residence (all *p* > 0.318). However, gender was not equally distributed across the experimental groups [χ^2^(3) = 12.2, *p* = 0.007]. Thus, gender was included in all subsequent analyses as a covariate.

**Figure 1:**
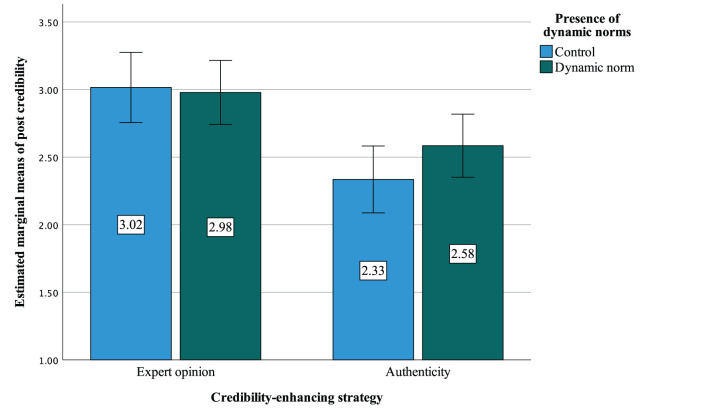
Testing the interaction effect of the credibility-enhancing strategy and the presence of dynamic norms on post credibility. The graph represents the estimated marginal means of post credibility that we compared with the two-way ANOVA (*N* = 386). Results indicated that expert opinion improved the credibility of the post; however, we did not observe any interaction effect of the credibility-enhancing strategies and dynamic norms.

We conducted a two-way ANOVA using the type of credibility-enhancing strategy and the presence of dynamic norms as independent variables and post credibility as dependent variable. Results indicated no interaction effect between the two independent variables [*F*_(1,379)_ = 1.30, *p* = 0.254, ${\text{η}}_{\text{p}}^{2}$ = 0.003]. However, a significant main effect of credibility-enhancing strategy was found [*F*_(1,379)_ = 18.6, *p* < 0.001, ${\text{η}}_{\text{p}}^{2}$ = 0.047] (see Figure 1). Expert opinion was perceived as more credible than authenticity management (*M*_*exp*_ = 2.98, *SD*_*exp*_ = 1.27 vs *M*_*role*_ = 2.48, *SD*_*role*_ = 1.16). However, we note that for both the expert opinion and authenticity management conditions, the credibility was below the medium scale point (3).

**Figure 2:**
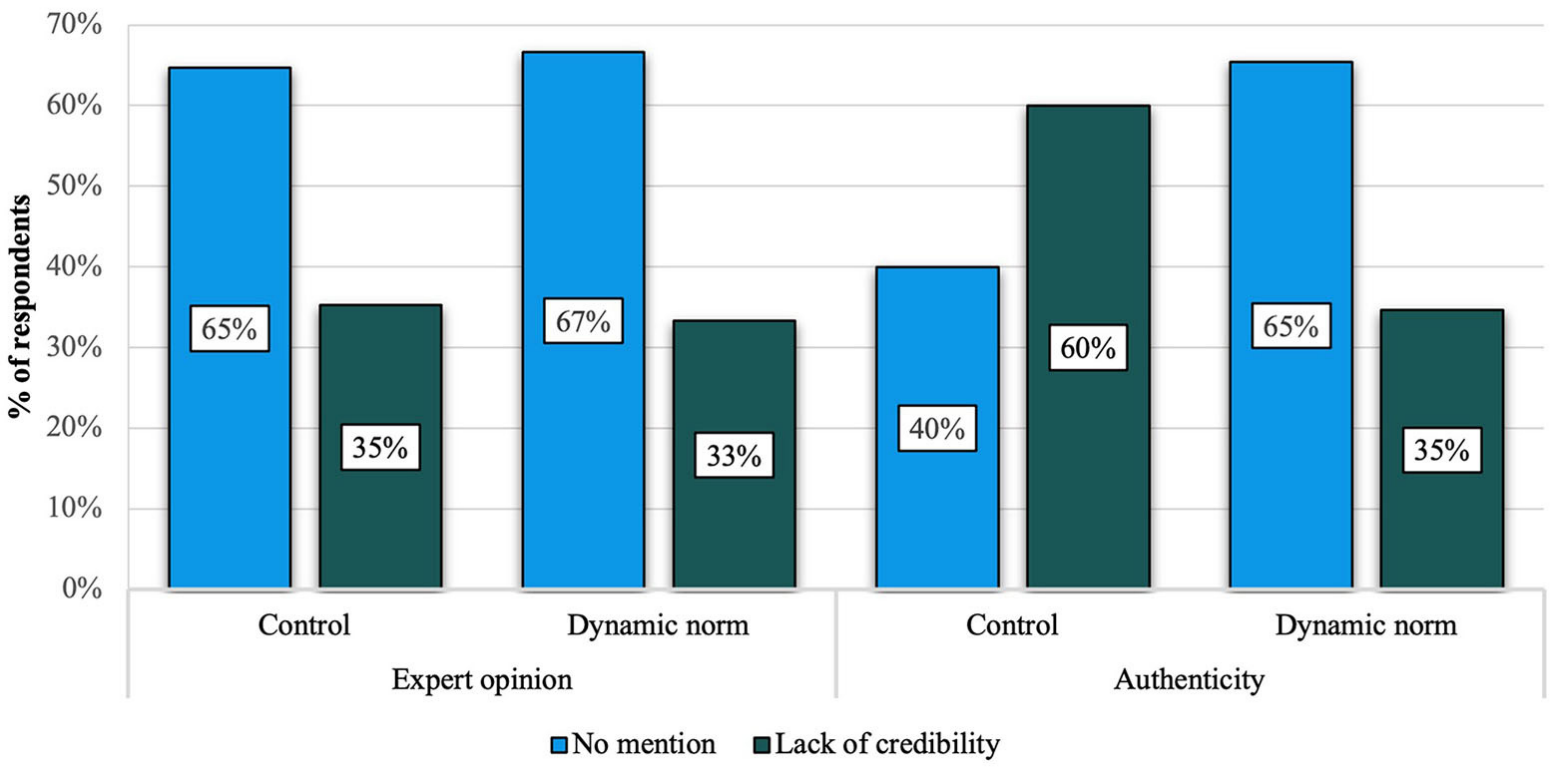
The interaction effect of the credibility-enhancing strategy and the presence of dynamic norms on the spontaneous mention of lack of credibility. The graph represents the percentage of respondents who mentioned/did not mention the lack of credibility by experimental condition (*N* = 386). Results indicated that dynamic norms improved the credibility of the post in the authenticity enhancing condition compared to its control; however, we did not observe such a salient difference regarding the expert opinion argument.

In the following step, a binary logistic regression was conducted to test the interaction effect of credibility-enhancing strategy and the presence of dynamic norms on the lack of credibility. Results indicated a significant interaction effect between the two predictor variables (*B* =−1.12, Wald χ^2^(1) = 6.56, *p* = 0.01) (see Figure 2). In the authenticity-enhancing condition, compared to the expert condition, when dynamic norms were present, it was less likely that respondents mention that the post lacked credibility. However, the same effect could not be observed when the post contained an expert opinion argument.

Finally, we examined whether the spontaneous mention of lack of credibility had an incremental predictive power on the persuasiveness of the post by conducting a hierarchical regression analysis. First, we calculated Spearman's rank correlation to assess the relationship between the two credibility measures. Results indicated a moderate negative correlation [*r*(386) = −0.552, *p* < 0.001). Then, we included the quantitative measure of post credibility as predictor variable and gender as covariate in the hierarchical regression model. Finally, spontaneously mentioned lack of credibility was added to the model. Results indicated a significant R^2^ change [${\text{R}}_{\text{change}}^{2}$ = 0.014, F_change_ (1, 370) = 14.2, *p* < 0.001]. Both credibility measures significantly predicted the persuasiveness of the post [post credibility: *b* = 0.706, *t*(370) = 18.5, *p* < 0.001, lack of credibility: *b* = −0.143, *t*(370) = −3.77, *p* < 0.001].

## 4. Discussion

Although attitudes toward sustainability are generally positive, those who adopt sustainable consumption habits in their everyday lives still represent a minority in the general population (Kumar, 2016; Chwialkowska, 2019). Moreover, people tend to underestimate the general concern about climate change (Sparkman et al., 2022). It is crucial to raise people's awareness of environmental issues and environmental risks (Li et al., 2022). While green influencers mostly attract followers who are interested in sustainability, non-green influencers can reach a wider, more heterogeneous audience. When encouraging sustainable consumption, non-green influencers have the potential to raise awareness among those who are not involved in living a sustainable lifestyle. On the other hand, non-green influencers face the issue of lack of credibility and potential greenwashing when posting about sustainability. In this study, we examined the effect of two different credibility-enhancing strategies and the presence of dynamic norms on the perceived credibility of the message. We also tested how the two different credibility measures that we assessed are related to the persuasiveness of the message.

First, the present results indicated a robust main effect of expert opinion argument vs. increasing authenticity on message credibility: including an expert opinion in the social media post led to increased message credibility. Thus, as proposed by Kruglanski et al. (2005), we provided empirical evidence that expert authority can be effectively conferred on other sources to increase message credibility regarding a sustainability topic. Moreover, the present results showed that referring to an expert opinion is more effective than the authenticity-increasing strategy that we tested. A possible explanation is that perceived similarities between the influencer and the audience also conveys authenticity (Abidin and Otis, 2016). In the present case, respondents might have perceived the influencer as being very different from them. Consequently, the lack of perceived similarity prevented them from engaging with the influencer and consider his efforts of promoting a sustainable message as being real and authentic. Besides, the study sample represented the general audience that can react in a different way compared to followers. For instance, authenticity plays a key role in the successful promotion of green lifestyle for followers (Chwialkowska, 2019), and followers perceive the influencer who promotes a sustainable product as more trustworthy with higher levels of intrinsic motives (Breves and Liebers, 2022). In contrast, the present results showed that a more rational approach of referring to expert opinion can lead to higher credibility perception for a general audience who are probably less involved with the influencer.

Second, somewhat surprisingly, no main effect of dynamic norms was found, or any interaction effects between the credibility-enhancing strategies and the presence of dynamic norms that would have affected the credibility of the message. One possible explanation of the present results might be the differences in the experimental settings: previous studies (see Sparkman and Walton, 2017; Sparkman et al., 2021) did not use an influencer as the source of message, nor did they include dynamic norms in a social media post or tested the effect of dynamic norms together credibility-enhancing strategies either. Furthermore, dynamic norms did not trigger the bandwagon heuristics either (“if others think it is important, I should think so, too”), probably because the perceived distance between the respondents and the influencer was large; thus, respondents did not feel the urge to identify with the trend described in the influencer's post.

Third, the present results showed that dynamic norms decreased the occurrence of spontaneous mentions of the lack of post credibility when included in the authenticity-enhancing post. On the other hand, the inclusion of dynamic norms in the expert opinion post did not affect the spontaneous mentions of the lack of credibility. We would like to underline the difference between the two credibility measures that we used in the study: the quantitative measure assessed the credibility of the post while the binary variable derived from the qualitative data assessed the perceived incredibility (lack of credibility) of the post. Thus, those who were exposed to the authenticity post with the dynamic norms might have not found the post more credible, instead, their perceptions of incredibility were attenuated. This result confirms our early assumption that non-green influencers communicating sustainable consumption may face a credibility issue: they are not sustainability experts, nor do they build a sustainable image as green influencers do; therefore, their sustainable communication can be perceived as greenwashing. Further confirming this assumption, post credibility scores were relatively low in the present study.

Regarding the interaction effect, the perceived similarity between the influencer and the respondent plays an important role in how authenticity (Audrezet et al., 2020) and dynamic norms (Sundar, 2008) affect credibility. This common underlying mechanism might have led to a positive synergic effect on credibility, explaining why dynamic norms decreased the lack of credibility when included in the authenticity-enhancing post, but they did not have the same effect when included in the expert opinion post. The effect of expert opinion on credibility is based on authority that does not require any engagement or perceived similarity with the influencer. Alternatively, as the authenticity-enhancing post was shorter than the expert opinion post, it is possible that the dynamic norm embedded in the expert opinion post was hard to perceive, and it was ignored without deeper processing.

Finally, it was demonstrated that besides the quantitative credibility measure, the lack of credibility of the post also significantly contributed to the explanation of the post's persuasiveness. These results are in line with previous research on the relationship between post credibility and persuasiveness (Martínez-López et al., 2020). Furthermore, the results also underline the importance of increasing the credibility perception of non-green influencers' sustainable communication. Indeed, lack of credibility can constitute an important barrier to the potential benefits of sustainable communication by non-green influencers.

### 4.1. Limitations

This study is not without limitations. First, we prioritized the ecological validity of the research. For this purpose, we used stimuli based on extracts of real influencer posts. More structured stimuli set, controlled for wording, length and other characteristics of the message could allow for the elimination of potential confounding variables. Second, a more structured stimuli set should also comprise a control condition without any credibility-enhancing strategies to assess the potential positive effects of credibility-enhancing strategies separately. Future research might consider adding other credibility-enhancing strategies to the research design. Third, the study sample represented a general audience. The study should be replicated among the followers of the influencer to explore possible differences in the effects. Fourth, persuasiveness was measured using one single item, which could be replaced with a more sophisticated scale. Furthermore, future studies should include different measures such as perceived similarity, authenticity, and source credibility to explore the underlying mechanisms of the effects demonstrated in this study. Finally, the generalizability of the present results is limited as we tested one sustainable message from one influencer. Future research should consider testing different sustainable messages from different influencers. Moreover, future research should consider testing the effect of messages posted by lifestyle influencers vs. green influencers.

## 5. Conclusion

In this study, we explored how non-green influencers can effectively increase the credibility of their posts about sustainable consumption. We tested two different credibility-enhancing strategies: referring to expert opinion and enhancing authenticity by expressing passion toward sustainability in a way that fits to the influencer. Besides, we also examined whether dynamic norms increase the effect of the credibility-enhancing strategies. According to our results, referring to expert opinion is a more effective credibility-enhancing strategy among general audience than the authenticity-increasing strategy. Moreover, dynamic norms included in the authenticity post decrease the perceived lack of credibility compared to the control condition. Results can be explained by the different underlying mechanisms of the credibility-enhancing strategies. Our study contributes to the sustainability communication literature by highlighting the importance of credibility-enhancing strategies in the case of non-green social media influencers.

## Data availability statement

The datasets presented in this study can be found in online repositories. The names of the repository/repositories and accession number(s) can be found at: Open Science Framework https://doi.org/10.17605/OSF.IO/5TZGX.

## Ethics statement

The studies involving human participants were reviewed and approved by the Institutional Review Board of ELTE, Hungary (no.2021/266-4). The patients/participants provided their written informed consent to participate in this study.

## Author contributions

ÁB, ÁZ, and GO contributed to the conception and study design. ÁB collected the data, performed the statistical analysis, and wrote the first version of the manuscript. All authors contributed to manuscript revision, read the final version, approved the publication of the manuscript, and agreed to be accountable for all aspects of the work.

## References

[B1] Abidin C. Otis M. (2016). “Influencers tell all. Unravelling Authenticity and Credibility in a Brand Scandal” in Blurring the Lines: Market-Driven and Democracy-Driven Freedom of Expression. ed. M. Edström, A. T. Kenyon, and E. M. Svensson. (Novi, MI: Nordicom), 153–161.

[B2] Appelman A. Sundar S. S. (2016). Measuring message credibility: construction and validation of an exclusive scale. J. Mass Commun. Q. 93, 59–79. 10.1177/1077699015606057

[B3] Audrezet A. Kerviler D. E. Guidry Moulard G. (2020). Authenticity under threat: when social media influencers need to go beyond self-presentation. J. Bus. Res. 117, 557–569. 10.1016/j.jbusres.2018.07.008

[B4] Breves P. Liebers N. (2022). Greenfluencing the impact of parasocial relationships with social media influencers on advertising effectiveness and followers' pro-environmental intentions. Environ. Commun. 77, 1–15. 10.1080/17524032.2022.2109708

[B5] Bruchmann K. Chue S. M. Dillon K. Lucas J. K. Neumann K. Parque C.. (2021). Social comparison information influences intentions to reduce single-use plastic water bottle consumption. Front. Psychol. 12:612662. 10.3389/fpsyg.2021.612662 34650463 PMC8506027

[B6] Campbell C. Farrell J. R. (2020). More than meets the eye: the functional components underlying influencer marketing. Business Horizons 63, 469–479. 10.1016/j.bushor.2020.03.003

[B7] Chan A. K. M. Nickson C. P. Rudolph J. W. Lee A. Joynt G. M. (2020). Social media for rapid knowledge dissemination: early experience from the COVID-19 pandemic. Anaesthesia 75, 1579–1582. 10.1111/anae.15057 32227594 PMC7228334

[B8] Chen Y. Awasthi A. K. Wei F. Tan Q. Li J. (2021). Single-use plastics: production, usage, disposal, and adverse impacts. Sci. Total Environ. 752, 141772. 10.1016/j.scitotenv.2020.141772 32892042

[B9] Chwialkowska A. (2019). How sustainability influencers drive green lifestyle adoption on social media: the process of green lifestyle adoption explained through the lenses of the minority influence model and social learning theory. Manage. Sust. Dev. 11, 33–42. 10.2478/msd-2019-0019

[B10] Cotte J. Coulter R. A. Moore M. (2005). Enhancing or disrupting guilt: the role of ad credibility and perceived manipulative intent. J. Bus. Res. 58, 361–368. 10.1016/S0148-2963(03)00102-4

[B11] Djafarova E. Rushworth C. (2017). Exploring the credibility of online celebrities' Instagram profiles in influencing the purchase decisions of young female users. Comput. Hum. Behav. 68, 1–7. 10.1016/j.chb.2016.11.009

[B12] Enli G. (2015). Mediated authenticity: How the Media Constructs Reality. New York, NY: Peter Lang.

[B13] Evans N. J. Phua J. Lim J. Jun H. (2017). Disclosing instagram influencer advertising: the effects of disclosure language on advertising recognition, attitudes, and behavioral intent. J. Int. Adv. 17, 138–149. 10.1080/15252019.2017.1366885

[B14] Fanghella V. D'Adda G. Tavoni M. (2019). On the use of nudges to affect spillovers in environmental behaviors. Front. Psychol. 10, 61. 10.3389/fpsyg.2019.00061 30761038 PMC6362870

[B15] Hoeken H. Hustinx L. (2003). “The relative persuasiveness of different types of evidence,” in Proceedings of the fifth conference of the International Society for the Study of Argumentation, ed. F. H. van Eemeren, J. A. Blair, C. A. Willard, and A. F. Snoeck Henkemans (Amsterdam: Sic Sat), 97–501.

[B16] Jin S. V. Muqaddam A. Ryu E. (2019). Instafamous and social media influencer marketing. MIP 37, 567–579. 10.1108/MIP-09-2018-0375

[B17] Johnstone L. Lindh C. (2018). The sustainability-age dilemma: a theory of (un)planned behaviour via influencers. J Consumer Behav 17, e127–e139. 10.1002/cb.1693

[B18] Kapoor P. S. Balaji M. S. Jiang Y. Jebarajakirthy C. (2022). Effectiveness of travel social media influencers: a case of eco-friendly hotels. J. Travel Res. 61, 1138–1155. 10.1177/00472875211019469

[B19] Kruglanski A. W. Raviv A. Bar-Tal D. Raviv A. Sharvit K. Ellis S.. (2005). Says who? Epistemic authority effects in social judgment. Adv. Exp. Soc. Psychol. 37, 345–392. 10.1016/S0065-2601(05)37006-7

[B20] Kumar P. (2016). State of green marketing research over 25 years (1990-2014): literature survey and classification. Market. Int. Planning 34, 137–158. 10.1108/MIP-03-2015-0061

[B21] Lee E. J. (2020). Authenticity model of (mass-oriented) computer-mediated communication: conceptual explorations and testable propositions. J. Comput. Mediated Commun. 25, 60–73. 10.1093/jcmc/zmz025

[B22] Li X. Liu Z. Wuyun T. (2022). Environmental value and pro-environmental behavior among young adults: the mediating role of risk perception and moral anger. Front. Psychol. 13, 771421. 10.3389/fpsyg.2022.771421 35222182 PMC8863658

[B23] Loschelder D. D. Siepelmeyer H. Fischer D. Rubel J. A. (2019). Dynamic norms drive sustainable consumption: norm-based nudging helps café customers to avoid disposable to-go-cups. J. Econ. Psychol. 75, 102146. 10.1016/j.joep.2019.02.002

[B24] Lou C. Yuan S. (2019). Influencer marketing: how message value and credibility affect consumer trust of branded content on social media. J. Int. Adv. 19, 58–73. 10.1080/15252019.2018.1533501

[B25] Marsh E. J. Yang B. W. (2021). One Believing Things That are not True: A Cognitive Science Perspective on Misinformation Misinformation and Mass Audiences. (Austin, TX: University of Texas Press), 15–34.

[B26] Martínez-López F. J. Anaya-Sánchez R. Esteban-Millat I. Torrez-Meruvia H. D'Alessandro S. Miles M.. (2020). Influencer marketing: brand control, commercial orientation and post credibility. J. Market. Manage. 36, 1805–1831. 10.1080/0267257X.2020.1806906

[B27] Matthes J. Wonneberger A. (2014). The skeptical green consumer revisited: testing the relationship between green consumerism and skepticism toward advertising. J. Adv. 43, 115–127. 10.1080/00913367.2013.834804

[B28] Ohanian R. (1990). Construction and validation of a scale to measure celebrity endorsers' perceived expertise, trustworthiness, and attractiveness. J. Adv. 19, 39–52. 10.1080/00913367.1990.10673191

[B29] Pittman M. Abell A. (2021). More trust in fewer followers: diverging effects of popularity metrics and green orientation social media influencers. J. Int. Market. 56, 70–82. 10.1016/j.intmar.2021.05.002

[B30] Rimal R. N. Storey J. D. (2020). Construction of meaning during a pandemic: the forgotten role of social norms. Health Commun. 35, 1732–1734. 10.1080/10410236.2020.1838091 33084409

[B31] Ryan R. M. Deci E. L. (2000). Self-determination theory and the facilitation of intrinsic motivation, social development, and well-being. Am. Psychol. 55, 68–78. 10.1037/0003-066X.55.1.68 11392867

[B32] Sparkman G. Geiger N. Weber E. U. (2022). Americans experience a false social reality by underestimating popular climate policy support by nearly half. Nat. Commun. 13, 4779. 10.1038/s41467-022-32412-y 35999211 PMC9399177

[B33] Sparkman G. Macdonald B. N. J. Caldwell K. D. Kateman B. Boese G. D. (2021). Cut back or give it up? The effectiveness of reduce and eliminate appeals and dynamic norm messaging to curb meat consumption. J. Environ. Psychol. 75, 101592. 10.1016/j.jenvp.2021.101592

[B34] Sparkman G. Walton G. M. (2017). Dynamic norms promote sustainable behavior, even if it is counternormative. Psychol. Sci. 28, 1663–1674. 10.1177/0956797617719950 28961062

[B35] Sundar S. S. (2008). “The main model: a heuristic approach to understanding technology effects on credibility,” in Digital Media, Youth, and Credibility, ed M. J. Metzger and A. J. Flanagin. (Cambridge, MA: The MIT Press), 73–100.

[B36] Uzunoglu E. Misci Kip S. (2014). Brand communication through digital influencers: leveraging blogger engagement. Int. J. Inf. Manage. 34, 592–602. 10.1016/j.ijinfomgt.2014.04.007

